# Incarceration of a Household Member and Hispanic Health Disparities: Childhood Exposure and Adult Chronic Disease Risk Behaviors

**DOI:** 10.5888/pcd10.120281

**Published:** 2013-05-02

**Authors:** Annie Gjelsvik, Dora M. Dumont, Amy Nunn

**Affiliations:** Author Affiliations: Dora M. Dumont, The Center for Prisoner Health and Human Rights, The Miriam Hospital, Providence, Rhode Island; Amy Nunn, Warren Alpert Medical School of Brown University, Division of Infectious Diseases, The Miriam Hospital, Providence, Rhode Island.

## Abstract

**Introduction:**

Incarceration of a household member has been linked to poor mental and behavioral health outcomes in children, but less is known about the health behaviors of these children once they reach adulthood.

**Methods:**

We analyzed data from 81,910 respondents to the 2009–2010 Behavioral Risk Factor Surveillance System to identify associations between the childhood experience of having a household member incarcerated and adult smoking status, weight status, physical activity, and drinking patterns. We used multivariable logistic regression to control for sex, age, education, and additional adverse childhood events in the whole population and in separate models for Hispanic, non-Hispanic white, and non-Hispanic black adults. We also assessed for having multiple risk behaviors.

**Results:**

People who lived with an incarcerated household member during childhood were more likely as adults than those who did not to engage in smoking (adjusted odds ratio [AOR] 1.50; 95% confidence interval [CI], 1.27–1.77) and heavy drinking (AOR 1.39; 95% CI, 1.03–1.87), after controlling for demographics and additional adverse childhood events. Exposure to incarceration in the household as a child among Hispanic adults was associated with being a smoker, being a heavy drinker, and having multiple risk behaviors and among white adults was associated with being a smoker and having multiple risk behaviors; among black adults there were no significant associations.

**Conclusion:**

Incarceration of a household member during childhood is associated with adult risk behaviors, and race/ethnicity may be a factor in this association.

## Introduction

The US incarceration rate surged starting in the 1970s and is now the highest in the world (1). Racial and ethnic disparities in incarceration rates accelerated at the same time. In 2009, the black male incarceration rate was 3,119 per 100,000, and the Hispanic male incarceration rate was 1,193 per 100,000; white males were incarcerated at a rate of 487 per 100,000 (2), a disparity that is explained by sociopolitical factors unrelated to crime (3,4). The number of children experiencing the incarceration of a household member has also grown dramatically. Results of a 2004 national survey by the Bureau of Justice Statistics showed that more than 50% of state prisoners and more than 60% of federal inmates had children younger than 18 years, and rates of parenthood were higher among black and Hispanic inmates than white inmates (5). Therefore, as these children reach adulthood, the effects on them will likely be higher for Hispanics and blacks than for whites.

People who have been incarcerated have a harder time acquiring stable housing, employment, education, and marriage partners (6,7), all of which are social determinants of health and may affect children living in the same household. Parental incarceration has been linked to poor mental health outcomes in children (8–11), and associations with children’s health may continue into adulthood. Although most studies of incarceration’s effects on community health have focused on infectious diseases (12), few (13–15) have addressed chronic disease risk factors. We hypothesized that an association exists between having an adverse childhood experience with incarceration of a household member and the following health behaviors: current smoking, binge or heavy drinking, being overweight or obese, and having no leisure-time physical activity. These behaviors have been identified as contributing to the leading causes of death in the United States (16).

## Methods

The Centers for Disease Control and Prevention’s (CDC’s) Behavioral Risk Factor Surveillance System (BRFSS) is an annual telephone-based survey administered by the 50 states, US territories, and Washington, DC. An optional adverse childhood events (ACEs) module was administered in 5 states in 2009 and in 8 states and the District of Columbia in 2010 (a combined state list of Arkansas, Hawaii, Louisiana, Maine, Nevada, New Mexico, Pennsylvania, Vermont, Washington, and Wisconsin). The response rates for these states in these years ranged from 47.0% (Pennsylvania) to 60.5% (Vermont) (17). The ACE module is based on questions from the Kaiser Permanente-CDC Adverse Childhood Experiences Study (18) and asks 8 questions relating to the respondent’s experiences before age 18.

Exposure during childhood to an incarcerated household member was measured by the question “Did you live with anyone who served time or was sentenced to serve time in a prison, jail, or other correctional facility?” In conformity with previous studies (19,20), responses of “don’t know” or “not sure” (<1% of responses) were considered no, and refusals were removed from the analysis. After removing data on respondents with missing information or who refused to answer the question (6.1% of adults in states that used the ACE module), the initial analytic sample consisted of 81,910 adults. Because the rates of having an incarcerated household member differ greatly by race/ethnicity, we stratified and assessed for effect measure modification by race/ethnicity for non-Hispanic whites, non-Hispanic blacks, and Hispanics. The outcomes were health behaviors associated with adverse health outcomes, as measured by the following: whether respondents were current smokers; whether they drank heavily, as calculated by whether the respondent had more than 2 drinks per day if male and 1 drink per day for female or whether they engaged in binge drinking (≥5 drinks for men and ≥4 for women on at least 1 occasion in the past 30 days); whether they were overweight (body mass index [BMI] of 25.0-29.9 kg/m^2^) or obese (BMI ≥30.0 kg/m^2^); and whether they reported no leisure-time physical activity in the past 30 days. Because these behaviors have been shown to cluster in the US adult population (21), we created an additional 3-level variable indicating 0 or 1, 2, and 3 or 4 of these behaviors.

Covariates were sex; age (18–25, 26–35, 36–45, 46–55, 55–65 and 66 or older); and education (no high school diploma, high school diploma or GED [general educational development], and some college or college degree). We did not include income as a covariate because more than 12% of respondents refused to respond or did not know their income. We controlled for ACEs other than living with an incarcerated household member, because people with 1 ACE are likely to have others (18). We created an ACE score using a method described elsewhere (19) while excluding the prison variable (range of 0–7), and we categorized the variable as having 0, 1, 2, or 3 to 7 additional ACEs.

We compared the distribution of sociodemographic characteristics and number of additional ACEs by having lived during childhood with an incarcerated household member using χ^2^ tests and calculated the distribution by age and race/ethnicity. We also used χ^2^ tests to compare the prevalence of health behaviors between adults who as a child had lived with an incarcerated household member and those who had not. We conducted 2 logistic regression models: the first with all covariates for the entire sample population and a second stratified by ethnicity. We used multinomial logit analysis to assess the association between experience of incarceration of a household member during childhood and a dependent ordinal health behavior variable with 3 levels (0 or 1, 2, and 3 or 4). For the health behaviors that had significant associations with experience of incarceration of a household member during childhood for white, Hispanic, or black adults we assessed for departure from additive effects. We used Stata version 11 (StataCorp LP, College Station, Texas) with survey commands to account for complex sampling design and weighting.

## Results

Few (6.5%) respondents in states using the ACE module in 2009 or 2010 had lived with an incarcerated household member during their childhood (Table 1). Those who did were less educated than those who had not (17% vs 6% had less than a high school education) and were more likely to have experienced other ACEs (68% had 3–7 other ACEs, compared with 19% of people who did not live with an incarcerated household member as a child). Childhood exposure to household incarceration was more common among the youngest age groups; 20% of respondents who had lived with an incarcerated household member were aged 18 to 25 compared with 8% of those who had not (Table 1). In all age groups, black and Hispanic adults had a higher prevalence of having experienced childhood exposure to an incarcerated household member, although the largest differences were seen in the younger age groups. (Figure).

**Table 1:** Selected Characteristics and Health Behaviors, by Having Lived During Childhood With an Incarcerated Household Member, Respondents (N = 81,910) of the Behavioral Risk Factor Surveillance System, 2009–2010

Characteristic	Did Not Live With Incarcerated Household Member, n (Weighted %) (n = 78,193)	Lived With Incarcerated Household Member, n (Weighted %) (n = 3,717)	*P* Value^a^
**Age, y**			
18–25	2,237 (8.0)	422 (20.0)	<.001
26–35	5,950 (15.8)	689 (30.0)	
36–45	10,173 (23.3)	699 (23.3)	
46–55	15,828 (19.8)	791 (14.6)	
56–65	18,806 (15.5)	653 (7.3)	
≥66	24,658 (17.6)	453 (4.8)	
**Sex**			
Female	48,393 (51.9)	2,218 (47.6)	.01
Male	29,800 (48.1)	1,499 (52.4)	
**Education**			
No high school diploma	5,598 (6.4)	590 (17.1)	<.001
High school diploma or GED	22,290 (29.6)	1,282 (36.9)	
Some college or college degree	50,181 (64.0)	1,841 (46.0)	
**Race/ethnicity**			
Non-Hispanic white	61,725 (82.9)	2,288 (68.5)	<.001
Non-Hispanic black	5,001 (6.3)	602 (15.7)	
Hispanic, all races	3,807 (4.5)	356 (8.1)	
Other, non-Hispanic	4,420 (4.4)	216 (3.5)	
Multi, non-Hispanic	2,297 (1.9)	218 (4.2)	
**Number of additional adverse childhood events**			
0	35,153 (45.4)	249 (5.4)	<.001
1	17,614 (23.3)	475 (11.9)	
2	9,584 (12.6)	579 (14.3)	
3–7	14,168 (18.7)	2,292 (68.4)	
**Health behaviors^b^**			
Current smoker	11,513 (17.5)	1,247 (38.7)	<.001
Overweight/obese	48,015 (65.0)	2,464 (66.7)	.33
No physical activity	19,158 (23.9)	1,080 (27.2)	.04
**Risky drinking**	9,915 (16.1)	712 (22.9)	<.001
Binge drinking	8,523 (14.7)	660 (22.0)	<.001
Heavy drinking	4,013 (4.8)	281 (8.2)	<.001
**Number of risk behaviors^c^**			
0 or 1	50,584 (65.8)	1,871 (49.5)	<.001
2	18,652 (26.6)	1,168 (36.3)	
3 or 4	4,507 (7.6)	505 (14.2)	

Abbreviation: GED, general educational development.

a Pearson χ^2^ tests were used to determine *P* values.

b See methods section for definitions of health behaviors.

c Number (0-4) out of current smoking, being overweight, having no physical activity, and risky drinking.

**Figure:**
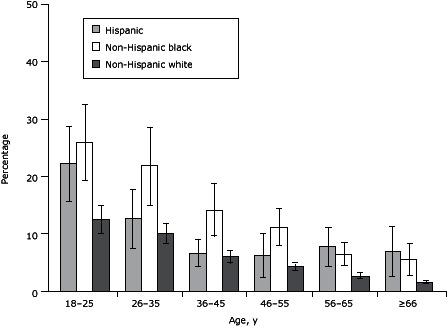
Percentage of adults who had a household member incarcerated during their childhood, by race/ethnicity and age group, Behavioral Risk Factor Surveillance System 2009–2010. Error bars represent 95% confidence intervals. **Table:** 

Age, y	% (95% Confidence Interval)		
	Hispanic	Non-Hispanic Black	Non-Hispanic White
18-25	22.2 (15.6-28.8)	26.0 (18.2-33.8)	12.5 (10.0-15.1)
26-35	12.7 (7.6-17.8)	21.8 (15.1-28.5)	10.2 (8.4-11.9)
36-45	6.6 (4.3-9.0)	14.2 (9.6-18.8)	6.0 (4.9-7.1)
46-55	6.3 (2.4-10.1)	11.2 (8.0-14.3)	4.3 (3.6-4.9)
55-65	7.7 (4.3-11.1)	6.5 (4.5-8.5)	2.7 (2.2-3.2)
≥66	6.9 (2.5-11.4)	5.6 (2.9-8.3)	1.6 (1.2-1.9)

People who had lived with an incarcerated household member had higher crude prevalence estimates for current smoking (39% vs 18%, *P* < .001) and heavy drinking (8% vs 5%; *P* < .001) but not for overweight or obesity or lack of physical activity (Table 1). Controlling for age, sex, education, race/ethnicity, and other ACEs, people who had a household member incarcerated during their childhood had higher odds of being current smokers (adjusted odds ratio [AOR], 1.50; 95% confidence interval [CI], 1.27–1.77) and higher odds of heavy drinking (AOR, 1.39; 95% CI 1.03–1.87) than those who did not (Table 2). The odds of being overweight or obese and reporting no physical activity were close to null. Respondents who had a household member incarcerated during their childhood had higher odds of having 2 versus 0 or 1 adverse health behaviors (AOR, 1.29; 95% CI, 1.09–1.54) and higher but nonsignificant odds of having 3 or 4 versus 0 or 1 health behaviors.

**Table 2:** Adjusted Odds^a^ of Engaging in Health Behaviors, Adults Who Lived As a Child With Someone Who Was Incarcerated^b^, Behavioral Risk Factor Surveillance System, 2009–2010

Health Behavior	Adjusted Odds Ratio (95% Confidence Interval)			
	All^a^	Hispanics^b^	Non–Hispanic White^b^	Non–Hispanic Black^b^
Current smoker	1.50 (1.27–1.77)	1.71 (1.07–2.76)	1.48 (1.20–1.83)	1.32 (0.88–1.97)
Overweight/obese	1.05 (0.88–1.24)	1.50 (0.86–2.64)	1.09 (0.89–1.33)	0.67 (0.44–1.03)
No physical activity	1.03 (0.87–1.23)	1.24 (0.74–2.08)	1.09 (0.88–1.36)	0.92 (0.62–1.36)
**Drinking behavior**				
Binge drinking	1.10 (0.89–1.34)	1.51 (0.87–2.60)	1.51 (0.88–2.59)	0.98 (0.55–1.71)
Heavy drinking	1.39 (1.03–1.87)	3.01 (1.45–6.25)	1.28 (0.87–1.88)	1.23 (0.59–2.56)
**Number of risk behaviors**				
2 vs 0 or 1	1.29 (1.09–1.54)	1.22 (0.71–2.07)	1.41 (1.14–1.75)	1.09 (0.72–1.66)
3 or 4 vs 0 or 1	1.22 (0.95–1.55)	1.99 (1.00–3.94)	1.20 (0.88–1.64)	0.85 (0.49–1.47)

a Controlled for age, sex, education, race/ethnicity, and other adverse childhood events.

b Compared with adults who did not live as a child with someone who was incarcerated.

When stratified by race/ethnicity, Hispanic adults who had a household member incarcerated during their childhood had higher odds of being current smokers (AOR, 1.71; 95% CI, 1.07–2.76), heavy drinking (AOR, 3.01; 95% CI, 1.45–6.25), and having 3 or 4 of the health behaviors versus 0 or 1 health behaviors (AOR, 1.99; 95% CI, 1.00–3.94) compared with those who did not. Non-Hispanic white adults who lived with an incarcerated household member had higher odds of current smoking (AOR, 1.48; 95% CI, 1.20–1.83) and having 2 versus 0 or 1 health behaviors (AOR, 1.41; 95% CI, 1.14–1.75) compared with those who did not. Among non-Hispanic black adults no significant associations were seen. 

There was evidence of effect measure modification of the association between exposure to living with an incarcerated household member during childhood and smoking and heavy drinking. Hispanic adults who were not exposed in childhood to living with an incarcerated household member had less than half the odds of heavy drinking compared with their similarly unexposed white counterparts (AOR, 0.44; 95% CI, 0.33–0.59) and white adults who were exposed in childhood to living with an incarcerated household member had essentially null odds compared with their white counterparts without the exposure (AOR 1.26; 95% CI 0.86–1.84). Yet, there were indications that the odds of heavy drinking were higher when both Hispanic and exposed to living with an incarcerated household member (AOR 1.66 95% CI 0.77–3.57) when compared with their white counterparts who were not similarly exposed in childhood, although it wasn’t statistically significant (Table 3). Although Hispanic adults who were not exposed in childhood to living with an incarcerated household member had about half the odds of current smoking compared with their similarly unexposed white counterparts (AOR, 0.51; 95% CI, 0.42–0.64), and white adults who were exposed in childhood to living with an incarcerated household member had higher odds compared with their white counterparts without the exposure (AOR, 1.50; 95% CI, 1.22–1.85), there were no significantly higher odds when both Hispanic and exposed to living with an incarcerated household member (AOR, 0.87; 95% CI, 0.55–1.37) when compared with their white counterparts who were not exposed in childhood.

**Table 3:** Adjusted Odds Ratios for Selected Health Behaviors Showing Interaction of Hispanic Ethnicity and Living With Someone During Childhood Who Was Incarcerated, Behavioral Risk Factor Surveillance System, 2009–2010

Characteristic	Smoking	Overweight/Obese	No Physical Activity	Heavy Drinking
White, no household prison exposure	1 [Reference]			
White, household prison exposure	1.50 (1.22–1.85)	1.10 (0.90–1.34)	1.11 (0.89–1.37)	1.26 (0.86–1.84)
Hispanic, no household prison exposure	0.51 (0.42–0.64)	1.21 (1.05–1.40)	1.17 (1.00–1.38)	0.44 (0.33–0.59)
Hispanic, household prison exposure	0.87 (0.55–1.37)	1.62 (0.95–2.79)	1.16 (0.71–1.90)	1.66 (0.77–3.57)

## Discussion

Hispanics have generally been excluded from the debate of whether incarceration exacerbates health disparities or, ironically, mitigates them by providing health care to medically underserved people of color (22–24). Our results indicate that this rapidly growing population may exhibit associations between household incarceration and adverse health behaviors that differ from non-Hispanic white and non-Hispanic black adults.

Our hypothesis that living with an incarcerated household member during childhood is associated with some adverse health behaviors later in life was partially proved, even after accounting for other coexisting ACEs. Why some behaviors are affected (smoking and drinking) and others (overweight/obesity and lack of physical activity) are not is unclear. The association with health behaviors was also evident among Hispanic and white but not black adults. Smoking prevalence is generally lower among Hispanics than among non-Hispanic whites (25), so the increased odds of smoking among Hispanic adults with childhood exposure to incarceration is concerning.

Incarceration and health are associated in complex ways. Prisoners have a worse health profile than the general population (13,14), and more than 50% suffer from poor mental health, substance dependence, or both (26,27). Little attention focused on Hispanic incarceration and its aftermath. Because of our small sample size, power was too weak to assess the association between race, incarceration, and health among Hispanics. Future studies may investigate how race mediates the Hispanic experience of both incarceration and health, especially for Hispanics who would be identified as black by US society regardless of how they self-identify. Race may mediate health among Hispanics as well as non-Hispanics, though identifying this is difficult because Hispanics are less likely to adopt US conceptions of racial categories. However, Hispanics who identify as black may be at increased risk of hypertension (28).

Previous studies have found associations between ACEs, including incarceration, and poor adult health behaviors and outcomes (19,29). To our knowledge, ours is the first to address childhood exposure to incarceration as a social determinant of health disparities, particularly vis-à-vis Hispanic health. Literature on incarceration’s effects on young children’s mental health is growing (8–11), but no other studies have addressed longer-term associations into adulthood. Likewise, studies of incarceration’s effects on community health have focused on infectious diseases (12) but, with a few exceptions (13–15), rarely address chronic diseases. Our analysis suggests that living with an incarcerated household member during childhood may be a social determinant in chronic disease risk behaviors.

Our study has limitations. Although the states using the ACE module are from different regions of the US and include varied demographic profiles, they may not be representative of the full national sample. We were unable to identify whether the effect varies depending on what type of household member (parent, sibling, or other) was incarcerated, length or frequency of incarceration, the type of offense (especially violent vs nonviolent), or the age of the respondent at the time of the incarceration. We were unable to control for the respondents’ own incarceration history in our analyses since the BRFSS, like most other nationally representative health data sets (30), does not collect this information.

In recent years, concern has grown regarding the fiscal and social costs of increasing incarceration, especially for nonviolent offenders. If the escalation of incarceration continues through the early years of this century, its public health effect will continue to grow as the children of those prisoners or former prisoners reach adulthood. Furthermore, any adverse health effects of this childhood experience will continue to contribute to health disparities for the foreseeable future, simply on the basis of disparities in the cumulative exposures. Public health practitioners should consider adding incarceration to the set of social determinants more generally known to shape health behaviors and outcomes in America.
